# Cardiovascular adverse event reporting profile of tirzepatide: a real-world pharmacovigilance analysis of heart failure, arrhythmias, and ischemic events

**DOI:** 10.3389/fphar.2026.1785129

**Published:** 2026-03-23

**Authors:** Daqiu Chen, Zixun Wang, Zhanxiong Xie, Yixing Chen, Shunxiang Luo, Shanghua Xu

**Affiliations:** 1 Department of Cardiology, Nanping First Hospital Affiliated to Fujian Medical University, Nanping, Fujian Province, China; 2 The Institute of Cardiovascular Sciences and Institute of Systems Biomedicine, School of Basic Medical Sciences, State Key Laboratory of Vascular Homeostasis and Remodeling, NHC Key Laboratory of Cardiovascular Molecular Biology and Regulatory Peptides, Beijing Key Laboratory of Cardiovascular Receptors Research, Health Science Center, Peking University, Beijing, China

**Keywords:** cardiovascular safety, FAERS, heart failure, pharmacovigilance, tirzepatide

## Abstract

**Background:**

Tirzepatide, a dual glucose-dependent insulinotropic polypeptide (GIP) and glucagon-like peptide-1 (GLP-1) receptor agonist, is highly effective for glycaemic control and weight reduction. However, its real-world cardiovascular adverse event reporting profile remains incompletely characterised.

**Methods:**

We conducted a retrospective pharmacovigilance study using the FDA Adverse Event Reporting System (FAERS) from the second quarter of 2022 (FDA approval of tirzepatide) through the third quarter of 2025. Disproportionality analysis was performed using the Reporting Odds Ratio (ROR) with a concurrent full-database comparator (all other drugs reported in the same quarterly files). Because consumers submitted 92.3% of reports, primary inference was drawn from analyses restricted to healthcare professional (HCP) reports (physicians and pharmacists). Subgroup analyses stratified by age, sex, and body weight were conducted; weight-stratified analyses were confined to reports with documented body weight.

**Results:**

A total of 103,693 unique tirzepatide reports were identified. Consumers accounted for 92.3% of submissions; body weight was documented in 5,881 reports (5.7%). In HCP-restricted analyses, tirzepatide was associated with persistently lower reporting odds for heart failure (ROR 0.18 [95% CI 0.07–0.43]). Similarly, no disproportionate reporting was detected for acute myocardial infarction (ROR 0.45 [0.19–1.08]), while remarkably, zero cases of angina or myocardial ischemia were reported in the HCP cohort. In contrast, no association was detected for atrial fibrillation (ROR 1.02 [0.58–1.80]) or tachycardia (ROR 1.01 [0.68–1.51]) in HCP reports. In weight-stratified analyses (full dataset, restricted to reports with documented weight), elevated RORs for tachycardia were observed in both weight groups (<95 kg: 1.91 [1.54–2.37]; ≥95 kg: 1.57 [1.20–2.05]); however, this finding was not replicated in the HCP-restricted analysis and should be interpreted with caution.

**Conclusion:**

In this real-world FAERS analysis using a concurrent full-database comparator and pre-specified stratification by reporter type, tirzepatide showed disproportionately low reporting of heart failure that was robust in HCP reports and across all subgroups. No reporting signal for atrial fibrillation, tachycardia, or ischemic events was detected after accounting for reporter bias. These findings provide pharmacovigilance evidence that complements the safety database of tirzepatide and generate hypotheses for ongoing cardiovascular outcome trials.

## Introduction

The global prevalence of obesity and type 2 diabetes mellitus (T2DM) has reached epidemic proportions, driving an urgent need for effective therapeutic interventions that address both metabolic dysregulation and associated cardiovascular risks (Shmygol et al., 2025; Campos et al., 2025). Glucagon-like peptide-1 receptor agonists (GLP-1RAs) have transformed the management of these conditions, demonstrating potent glucose-lowering and weight-loss effects alongside established cardiovascular benefits (Solini et al., 2023; Sheth et al., 2025). Building on this success, tirzepatide, a novel first-in-class dual glucose-dependent insulinotropic polypeptide (GIP) and GLP-1 receptor agonist, has emerged as a transformative agent. By synergistically activating both incretin receptors, tirzepatide has demonstrated superior efficacy in glycemic control and body weight reduction compared to selective GLP-1RAs in the landmark SURPASS (Study of Tirzepatide in Participants With Type 2 Diabetes) and SURMOUNT (Study of Tirzepatide in Participants With Obesity) clinical trial programs (Samms et al., 2022; Novikoff and Muller, 2023).

Despite its promising metabolic profile, the cardiovascular safety of tirzepatide remains a subject of intense clinical interest and ongoing investigation. While GLP-1RAs are known to reduce the risk of major adverse cardiovascular events (MACE) in high-risk populations (Badve et al., 2025), they are also associated with a consistent increase in resting heart rate, raising theoretical concerns about potential arrhythmogenic risks or deleterious hemodynamic effects over the long term (Greco et al., 2022). The initial data from the Phase three trial of tirzepatide showed a dose-dependent increase in heart rate (Yang et al., 2024). Although a pre-specified meta-analysis of the SURPASS trials suggested no increase in MACE risk (Sattar et al., 2022), these trials were not primarily powered to adjudicate specific cardiovascular outcomes such as heart failure or atrial fibrillation, and patients with unstable cardiovascular conditions were often excluded.

Furthermore, recent evidence has sparked a paradigm shift regarding the potential of incretin-based therapies in heart failure management. The STEP-HFpEF trial (Semaglutide Treatment Effect in People with obesity and HFpEF) recently demonstrated that semaglutide significantly reduced symptoms and physical limitations in patients with heart failure with preserved ejection fraction (HFpEF) and obesity, suggesting a direct cardioprotective role beyond weight loss (Schou et al., 2024; Kosiborod et al., 2023). However, whether tirzepatide, with its additional GIP component, confers similar or distinct cardiovascular benefits—or risks—in a broader, unselected real-world population remains largely unknown. Post-marketing surveillance is crucial to bridge the gap between controlled clinical trials and real-world clinical practice, where patient heterogeneity regarding age, comorbidities, and polypharmacy is substantially higher (Wang M. et al., 2025; Hunter Gibble et al., 2025).

Therefore, this study aimed to characterize the real-world cardiovascular adverse event reporting profile of tirzepatide using the FAERS database from 2022 to 2025. Specifically, we sought to: (1) assess the reporting frequency of major cardiovascular events, including heart failure, atrial fibrillation, myocardial infarction, angina, myocardial ischemia, and tachycardia, relative to a concurrent full-database comparator; (2) evaluate whether these reporting patterns are modified by baseline factors such as body weight, age, and sex; and (3) analyze longitudinal trends to determine the stability of any observed associations over time.

## Methods

### Data source and acquisition

This retrospective pharmacovigilance study utilized data from the FAERS, a publicly available database designed to support the FDA’s post-marketing safety surveillance program (Potter et al., 2025). We extracted quarterly data files (ASCII format) spanning from the second quarter of 2022 (corresponding to the FDA approval of tirzepatide in May 2022) to the third quarter of 2025.

The raw data files included demographic information (DEMO), drug information (DRUG), and reaction information (REAC). To ensure data integrity and reproducibility, all data processing and statistical analyses were performed using R software (version 4.4.1, Foundation for Statistical Computing, Vienna, Austria).

### Data processing and deduplication

Given the voluntary nature of FAERS reporting, duplicate entries are common (e.g., initial reports followed by follow-up reports). We implemented a rigorous deduplication process recommended by the FDA.

1. Case Identification: Reports were grouped by CASEID.
2. Selection Criteria: For reports with the same CASEID, we retained the record with the most recent fda_dt (FDA receipt date). If the dates were identical, the record with the highest PRIMARYID was selected as the final unique case to capture the most complete information.

## Drug identification

We identified cases associated with tirzepatide using a text-mining approach. The DRUGNAME column was searched using a case-insensitive regular expression query to capture both generic and brand names, including: “tirzepatide”, “Mounjaro”, “Zepbound”, and the research code “LY3298176.”

**FIGURE 1:**
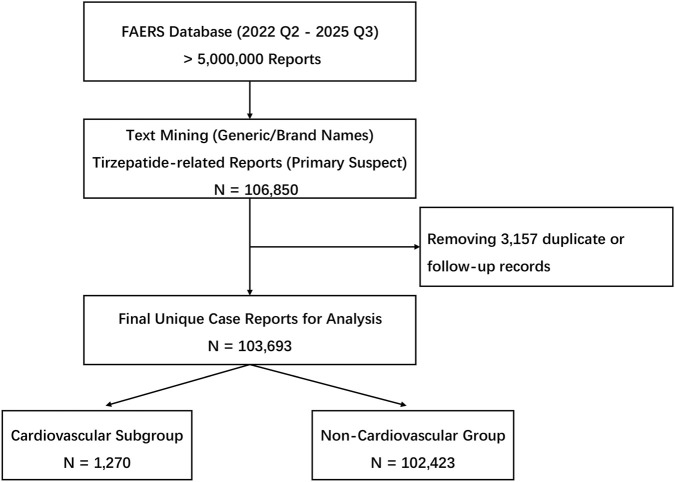
Flow diagram of case selection. Flow diagram illustrating the case selection process from the FDA Adverse Event Reporting System (FAERS) database, covering reports from the second quarter of 2022 to the third quarter of 2025. The final analytical cohort comprised 103,693 unique reports where tirzepatide was listed as the primary suspect drug.

To minimize confounding bias and focus on drug-induced events, we restricted the analysis to reports where tirzepatide was listed as the “Primary Suspect” (PS) drug (Figure 1). Concomitant or interacting drugs were excluded from the exposure definition.

### Definition of adverse events

Adverse events (AEs) were coded using the Medical Dictionary for Regulatory Activities (MedDRA). We focused on specific cardiovascular outcomes. Based on Standardised MedDRA Queries (SMQ) and clinical relevance, we defined the following events of interest using keyword matching on the Preferred Term (PT) level.

1. Heart Failure: Identified by terms including “Heart failure”, “Cardiac failure”, “Pulmonary oedema”, and “Ejection fraction decreased”.
2. Atrial Fibrillation: Identified by the term “Atrial fibrillation”.
3. Tachycardia: Identified by terms including “Tachycardia”, “Sinus tachycardia”, and “Heart rate increased”.
4. Myocardial Infarction: Identified by terms including “Myocardial infarction” and “Acute myocardial infarction”.
5. Angina: Identified by terms including “Angina pectoris” and “Unstable angina”.
6. Myocardial Ischemia: Identified by terms including “Myocardial ischaemia” and “Ischaemia myocardial”.

### Statistical analysis

Baseline characteristics were summarized for the overall cohort and stratified by cardiovascular status. Continuous variables: Age was presented as mean ± standard deviation (SD) as it followed a normal distribution. Weight was presented as median [interquartile range, IQR: Q1–Q3] due to its skewed distribution and the presence of outliers. Categorical variables: Sex, reporting year, and reporter type were expressed as counts and percentages (%). Hypothesis Testing: Differences between groups were assessed using the T-test for normally distributed continuous variables, the Mann-Whitney U test for non-normally distributed variables (weight), and the Chi-square test for categorical variables.

To evaluate the strength of the association between tirzepatide and specific cardiovascular events, we calculated the Reporting Odds Ratio (ROR) based on a standard two-by-two contingency table (Wang L. et al., 2025).

The formulas are as follows:

$$ROR=\frac{ad}{bc}$$

The 95% Confidence Interval (95% CI) for the ROR was calculated as:

$$95\%\;CI=e^{\ln\left(ROR\right)\pm 1.96\sqrt{\frac{1}{a}+\frac{1}{b}+\frac{1}{c}+\frac{1}{d}}}$$

where:

- Number of reports containing the target event for tirzepatide;
- Number of reports containing all other adverse events for tirzepatide;
- Number of reports containing the target event for all other drugs (background comparator);
- Number of reports containing all other adverse events for all other drugs (background comparator).

We utilized the full FAERS database (concurrent comparator) to derive the background odds (c/d). This approach adheres to standard pharmacovigilance practices by comparing the reporting frequency of an event for tirzepatide against the concurrent reporting frequency of the same event for all other medications in the database.

Furthermore, to ensure robust estimates in subgroup analyses, we applied stratum-specific comparators. For analyses stratified by age, sex, weight, and reporter type (e.g., all reports vs. HCP reports only), the RORs were calculated using the subset of the full database corresponding to that specific subgroup (e.g., tirzepatide cases in males were compared against all other drug cases in males) to minimize confounding by demographic characteristics.

Subgroup and Trend Analysis: 1. Subgroup Analysis: We stratified the ROR calculations by age (<65 years vs. ≥65 years), sex (Male vs. Female), and body weight (<95 kg vs. ≥95 kg) to assess the consistency of safety signals across different patient profiles. The 95 kg cutoff was chosen based on the median weight of the study population to ensure balanced sample sizes for subgroup analysis. 2. Time Trend Analysis: We analyzed the longitudinal trend of reported events from 2022 to 2025 to assess whether safety signals were stable or changing over time.

A signal was considered significant if the lower bound of the 95% Confidence Interval (CI) was greater than 1.0 (indicating elevated reporting odds) or the upper bound was less than 1.0 (indicating an inverse association). Data visualization was performed using the ggplot2 package in R.

## Results

### Study population and descriptive characteristics

The selection process for relevant cases is illustrated in Figure 1. From the initial retrieval of 106,850 reports related to tirzepatide in the FAERS database (Q2 2022 – Q3 2025), a total of 103,693 unique cases were included in the final analysis after removing duplicates. Among these, 1,270 cases were identified as having cardiovascular (CV) events, while the remaining 102,423 cases served as the Non-Cardiovascular Group (control group).

The demographic characteristics of the study population are summarized in Table 1. The mean age of the overall cohort was 53.8 ± 13.6 years, with no statistically significant difference observed between the CV Event Group and the Control Group (54.8 ± 15.1 vs. 53.8 ± 13.6 years, *P* = 0.0632). In terms of body weight, patients in the CV Event Group were slightly heavier than those in the Control Group (Median [IQR]: 94.0 kg [80.0–112.0] vs. 91.8 kg [78.3–108.0], *P* = 0.038). Regarding sex distribution, while females constituted the majority in both groups, the proportion of male patients was significantly higher in the CV Event Group compared to the Control Group (26.4% vs. 20.2%, *P* < 0.001). The number of reports increased substantially over the study period, with the majority filed in 2024 and 2025.

**TABLE 1:** Demographic and clinical characteristics of patients with tirzepatide-related reports in the FAERS database (2022 Q2 – 2025 Q3).

Characteristic	Total (N = 103,693)	Control group (N = 102,423)	CV event group (N = 1,270)	*P-value*
Age (Years), mean (SD)	53.8 (13.6)	53.8 (13.6)	54.8 (15.1)	0.0632
Weight (kg) median [Q1, Q3]	92.0 [78.5, 108.0]	91.8 [78.3, 108.0]	94.0 [80.0, 112.0]	0.038
Missing	97,812 (94.3%)	96,862 (94.6%)	950 (74.8%)	​
Gender	​	​	​	<0.001
Female	64,349 (62.1%)	63,590 (62.1%)	759 (59.8%)	​
Male	21,040 (20.3%)	20,705 (20.2%)	335 (26.4%)	​
Reporting year	​	​	​	<0.001
2022	4,966 (4.8%)	4,925 (4.8%)	41 (3.2%)	​
2023	20,737 (20.0%)	20,563 (20.1%)	174 (13.7%)	​
2024	32,980 (31.8%)	32,552 (31.8%)	428 (33.7%)	​
2025	45,010 (43.4%)	44,383 (43.3%)	627 (49.4%)	​
Reporter type	​	​	​	<0.001
Consumer	95,681 (92.3%)	94,613 (92.4%)	1,068 (84.1%)	​
Other	4,385 (4.2%)	4,297 (4.2%)	88 (6.9%)	​
Pharmacist	1,109 (1.1%)	1,077 (1.1%)	32 (2.5%)	​
Physician	2,518 (2.4%)	2,436 (2.4%)	82 (6.5%)	​

Abbreviations: CV, cardiovascular; FAERS, FDA, adverse event reporting system; SD, standard deviation; IQR, interquartile range (25th–75th percentiles).

## Signal detection of specific cardiovascular outcomes

Disproportionality analysis, utilizing the full FAERS database as the background comparator, characterized the reporting profile of tirzepatide regarding specific cardiovascular outcomes (Table 2).

**TABLE 2:** Signal detection of cardiovascular outcomes (stratified by reporter type).

Adverse event	Count (All)	Frequency (All)	ROR (All) [95% CI]	Count (HCP)	Frequency (HCP)	ROR (HCP) [95% CI]
Heart failure	70	0.07%	0.08 [0.07–0.11]	5	0.20%	0.18 [0.07–0.43]
Atrial fibrillation	125	0.13%	0.29 [0.24–0.35]	12	0.49%	1.02 [0.58–1.80]
Tachycardia	595	0.60%	0.62 [0.57–0.67]	24	0.97%	1.01 [0.68–1.51]
Acute MI	151	0.15%	0.32 [0.27–0.37]	5	0.20%	0.45 [0.19–1.08]
Angina	14	0.01%	0.14 [0.08–0.23]	0	0	NC
Myocardial ischemia	3	<0.01%	0.12 [0.04–0.36]	0	0	NC

“All” represents the primary analysis utilizing the full dataset (including both consumer and healthcare professional reports). “HCP” represents the sensitivity analysis restricted strictly to reports submitted by healthcare professionals (e.g., physicians and pharmacists). “NC” (Not Calculable) indicates that the ROR, and its 95% CI, could not be computed due to zero observed events in the respective group. Abbreviations: CI, confidence interval; HCP, healthcare professional; MI, myocardial infarction; NC, not calculable; ROR, reporting odds ratio.

In the full dataset, tirzepatide was associated with lower reporting odds across all assessed cardiovascular events, including heart failure (ROR 0.08 [95% CI 0.07–0.11]), atrial fibrillation (ROR 0.29 [0.24–0.35]), tachycardia (ROR 0.62 [0.57–0.67]), acute myocardial infarction (ROR 0.32 [0.27–0.37]), angina (ROR 0.14 [0.08–0.23]), and myocardial ischemia (ROR 0.12 [0.04–0.36]).

In the sensitivity analyses restricted to healthcare professional (HCP) reports, the reporting profile demonstrated notable divergences. Tirzepatide remained associated with persistently lower reporting odds for heart failure (ROR 0.18 [0.07–0.43]). Regarding ischemic outcomes, no significant disproportionate reporting was detected for acute myocardial infarction (ROR 0.45 [0.19–1.08]). Strikingly, there were zero reported cases of either angina or myocardial ischemia in the HCP cohort, rendering the respective RORs not calculable (NC) but heavily underscoring the lack of any ischemic safety signal in clinical practice. In contrast to the findings in the full dataset, no significant association was detected for atrial fibrillation (ROR 1.02 [0.58–1.80]) or tachycardia (ROR 1.01 [0.68–1.51]) after accounting for reporter bias in HCP reports.

### Subgroup analysis and robustness check

**FIGURE 2:**
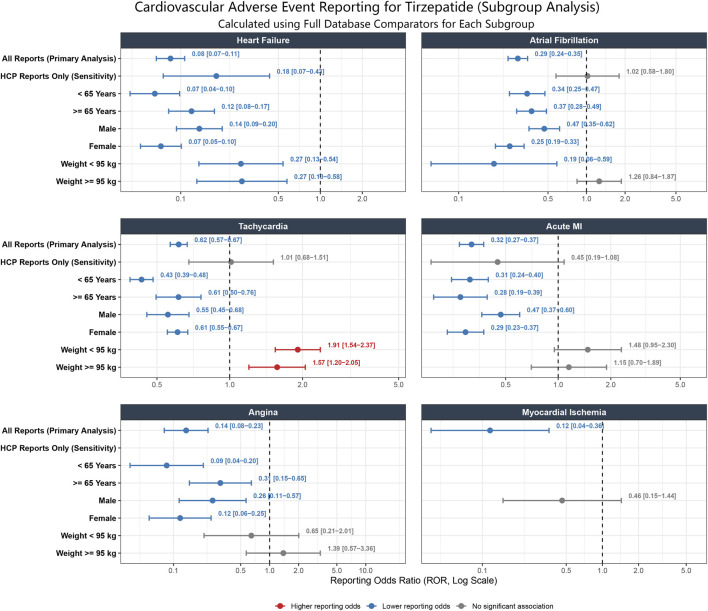
Subgroup analysis of cardiovascular adverse event reporting for tirzepatide. Forest plot displaying Reporting Odds Ratios (ROR) with 95% confidence intervals for six cardiovascular outcomes: heart failure, atrial fibrillation, tachycardia, acute myocardial infarction, angina, and myocardial ischemia. Blue indicates lower reporting odds, red indicates higher reporting odds, and grey indicates no significant association. Weight-stratified analyses are based on the 5,881 reports (5.7% of total) with documented body weight. Abbreviations: FAERS, FDA Adverse Event Reporting System; ROR, Reporting Odds Ratio.

To assess whether the observed signals were influenced by reporter bias or baseline demographic differences, we performed subgroup analyses stratified by data source (All Reports vs. HCP Reports Only), age, sex, and weight (Figure 2).

Body weight was documented in only 5,881 of 103,693 reports (5.7% of the total cohort), corresponding to a missing rate of 94.3%. All weight-stratified analyses presented below are confined to this limited subset and may not be generalizable to the entire population.

The lower reporting odds for heart failure remained consistently observed across all strata. Specifically, significantly lower reporting odds were robustly maintained in the sensitivity analysis restricted to HCP Reports Only (ROR 0.18 [95% CI: 0.07–0.43]) and across both weight categories, with identical RORs of 0.27 observed in both the lower weight (<95 kg: ROR 0.27 [0.13–0.54]) and higher weight (≥95 kg: ROR 0.27 [0.13–0.58]) groups.

Similarly, the reporting profiles for ischemic events (acute myocardial infarction, angina, and myocardial ischemia) remained consistently low or neutral across the evaluated subgroups. In the HCP-restricted sensitivity analysis, acute myocardial infarction shifted to neutrality (ROR 0.45 [0.19–1.08]), while angina and myocardial ischemia recorded zero events, rendering their RORs not calculable but heavily underscoring a lack of ischemic safety signals. In the weight-stratified analyses, the reporting odds for acute myocardial infarction also shifted to neutrality in both the lower (<95 kg: ROR 1.48 [0.95–2.30]) and higher (≥95 kg: ROR 1.15 [0.70–1.89]) weight groups, with confidence intervals crossing the null value. A similar neutral trend was observed for angina across these weight strata (<95 kg: ROR 0.65 [0.21–2.01]; ≥95 kg: ROR 1.39 [0.57–3.36]), whereas myocardial ischemia recorded zero cases in the weight subset.

In contrast, the signals for arrhythmias exhibited divergence from the trend observed in All Reports. For atrial fibrillation, the apparent lower reporting odds observed in the primary analysis shifted to neutrality in the HCP Reports Only analysis (ROR 1.02 [0.58–1.80]) and in the higher weight group (≥95 kg: ROR 1.26 [0.84–1.87]), with confidence intervals including the null value.

Most notably, regarding tachycardia, while All Reports suggested a lower reporting probability, the weight-stratified analyses revealed significant elevated reporting odds in both weight categories (<95 kg: ROR 1.91 [1.54–2.37]; ≥95 kg: ROR 1.57 [1.20–2.05]). This unmasking of the tachycardia signal in patients with reported weight is consistent with the known chronotropic effects of GLP-1 receptor agonists and suggests that the lower reporting odds observed in All Reports were likely an artifact of under-reporting in incomplete case reports.

#### General safety landscape

**FIGURE 3:**
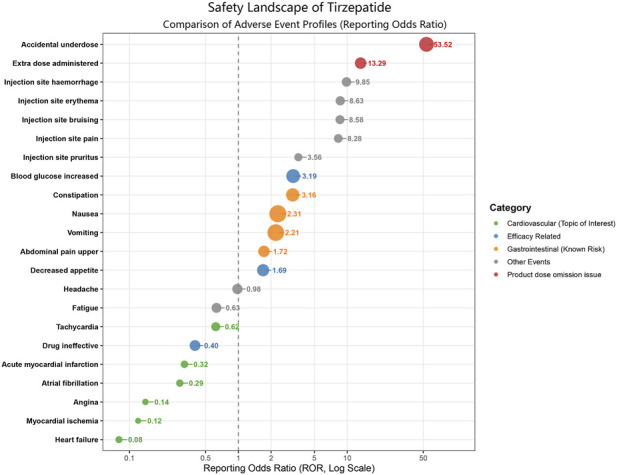
Safety landscape of adverse events associated with tirzepatide. Bubble plot comparing ROR (log scale) and case frequency. Note: This figure is derived from all reports, of which 92.3% were submitted by consumers; cardiovascular signals observed here do not persist in HCP-restricted analyses (see Table 2). Abbreviations: ROR, Reporting Odds Ratios.

To contextualize the cardiovascular outcomes within the broader safety profile of tirzepatide, we generated a landscape bubble plot comparing CV events with the top reported adverse events (Figure 3).

The safety landscape was predominantly characterized by administration-related errors and gastrointestinal (GI) disorders. Events such as accidental underdose (ROR 53.52) and extra dose administered exhibited the strongest disproportionality signals, alongside known GI effects like nausea and vomiting, representing the primary burden of adverse effects. This visualization highlights a critical safety context: the reporting frequency of these administration and tolerability issues substantially outweighs that of cardiovascular events.

In sharp contrast, all evaluated cardiovascular outcomes clustered distinctly in the zone of lower reporting odds (left side of the plot, ROR <1). Heart failure (ROR 0.08) and the ischemic events—myocardial ischemia (ROR 0.12) and angina (ROR 0.14)—clustered tightly at the extreme lower end of the spectrum. Similarly, atrial fibrillation (ROR 0.29), acute myocardial infarction (ROR 0.32), and tachycardia (ROR 0.62) also fell within this low-reporting zone. This contrast underscores that cardiovascular events were reported at lower frequencies than the database average in the full dataset, whereas administration-related and gastrointestinal events dominated the reporting landscape.

### Longitudinal trends

**FIGURE 4:**
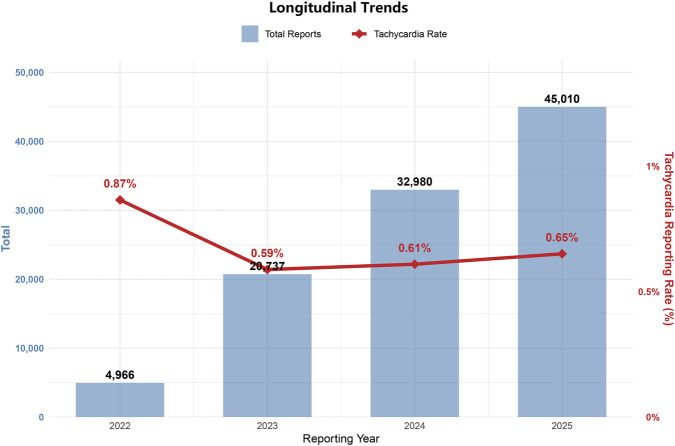
Longitudinal trends in reporting volume and tachycardia signal. The figure shows the exponential increase in the total number of tirzepatide-related reports (bars, left axis) alongside the stable proportional reporting rate of tachycardia (line, right axis) from 2022 through 2025.

Given the rapid uptake of tirzepatide, we analyzed the longitudinal reporting trends to assess potential cumulative risks (Figure 4). The total number of tirzepatide-related reports surged exponentially from 4,966 in 2022 to 45,010 in 2025.

Despite this dramatic increase in volume, the proportional reporting rate of tachycardia remained highly stable over the 4-year period, fluctuating narrowly between 0.59% and 0.87%. This stability is consistent with a pharmacological class effect rather than cumulative toxicity.

## Discussion

This study, analyzing over 100,000 pharmacovigilance reports from the FDA FAERS database, provides a comprehensive real-world assessment of the cardiovascular adverse event reporting profile of tirzepatide. A critical methodological consideration in our analysis is the overwhelming proportion of consumer-submitted reports (92.3%). Because consumer reports are highly susceptible to subjective interpretation—where physiological palpitations may be inaccurately coded as “tachycardia,” and complex conditions like heart failure may be underreported due to a lack of formal medical diagnosis—our primary clinical inferences are drawn from sensitivity analyses restricted to healthcare professional (HCP) reports. Utilizing a concurrent full-database comparator, our HCP-restricted analysis reveals a reporting profile characterized by persistently lower reporting odds for heart failure (ROR 0.18, 95% CI: 0.07–0.43). Concurrently, the signals for atrial fibrillation (ROR 1.02, 95% CI: 0.58–1.80) and tachycardia (ROR 1.01, 95% CI: 0.68–1.51) shifted to the null in the HCP cohort. Similarly, ischemic events including acute myocardial infarction shifted to neutrality (ROR 0.45, 95% CI: 0.19–1.08), while remarkably, angina and myocardial ischemia recorded zero cases in the HCP subset. These findings suggest no disproportionate reporting of these arrhythmic and ischemic events when restricted to medically verified cases.

It is imperative to emphasize that FAERS disproportionality analyses reflect reporting patterns rather than true reductions or elevations in clinical risk (Donati et al., 2025; Irizarry et al., 2025). Therefore, a Reporting Odds Ratio (ROR) of less than one must not be misconstrued as clinical “cardioprotection.” Lower RORs in spontaneous reporting systems frequently result from competing adverse event reporting. As demonstrated in our landscape analysis (Figure 3), the tirzepatide reporting profile is heavily dominated by gastrointestinal adverse events and administration-related errors, which mathematically depress the proportional reporting of cardiovascular events (Garvey et al., 2023; Rubino et al., 2025). Furthermore, these lower reporting odds may be significantly influenced by confounding by indication and channeling bias, wherein patients with severe pre-existing cardiovascular disease or advanced heart failure might be preferentially prescribed alternative therapies or closely monitored, altering the spontaneous reporting dynamic (Sendaydiego et al., 2024; Grubic et al., 2025).

Nevertheless, the persistently lower reporting odds for heart failure, which remained robust in the HCP-restricted analysis, is a notable finding that aligns with recent paradigm-shifting clinical trials. For example, the STEP-HFpEF trial demonstrated that semaglutide significantly improved outcomes in patients with heart failure with preserved ejection fraction (HFpEF) (Kosiborod et al., 2023; Kosiborod et al., 2024). Our real-world data suggest that the dual GIP/GLP-1 agonist tirzepatide does not exhibit a safety signal for heart failure, which is consistent with the hypothesized underlying mechanisms that extend beyond weight reduction. Specifically, the additional activation of GIP receptors by tirzepatide may confer unique advantages in adipose tissue and the vasculature, hypothesized to enhance the “buffering capacity” of white adipose tissue, preventing myocardial lipotoxicity (Campbell et al., 2022; Mestres-Arenas et al., 2026). This concept is further reinforced by our findings regarding ischemic outcomes. The complete absence of angina and myocardial ischemia reports in the HCP-restricted cohort, alongside neutral signals for acute myocardial infarction, strongly complements the proposed anti-atherosclerotic potential of GLP-1/GIP dual agonism to improve endothelial function and plaque stability (Pujadas et al., 2022).

Regarding atrial fibrillation (AF) and tachycardia, the inverse associations observed in the full dataset were not replicated in HCP-restricted analyses. This divergence strongly suggests that the initial low reporting was an artifact of consumer reporting patterns rather than a true clinical absence of the events. Furthermore, our weight-stratified analyses revealed elevated reporting odds for tachycardia in both the lower (<95 kg: ROR 1.91) and higher (≥95 kg: ROR 1.57) weight groups. However, these findings must be interpreted with extreme caution. Body weight data were missing in 94.3% of the total cohort, meaning these stratified analyses were confined to a highly selected subset of only 5,881 reports. Patients with meticulously documented weight in spontaneous reporting systems often represent cases undergoing intensive clinical monitoring. In such meticulously documented sub-cohorts, expected pharmacological effects—such as the known dose-dependent chronotropic effect of GLP-1 receptor agonists (increases of two to four beats per minute documented in the SURPASS and SURMOUNT trials)—are more likely to be “unmasked” and captured (Yang et al., 2024; Ayesh et al., 2024). We strictly refrain from drawing mechanistic conclusions regarding the weight-related heterogeneity of atrial fibrillation based on this incomplete subgroup reporting, as the unclear denominators and extensive missing data preclude reliable biological inferences.

Although elevated heart rate is a traditional cardiovascular risk factor, our longitudinal trend analysis (Figure 4) indicates this signal is non-progressive. While a visibly higher reporting rate of tachycardia was observed in 2022, this initial peak is characteristic of the “Weber effect”—a well-documented pharmacovigilance phenomenon where adverse event reporting surges in the first year following a drug’s approval due to heightened clinical vigilance and novelty (Carriot et al., 2021; Gérard et al., 2022).

This study has major limitations inherent to spontaneous reporting systems. First, disproportionality analysis measures statistical associations in reporting frequencies and does not establish definitive causality or quantify absolute risk. Second, the lack of a denominator (total exposed population) prevents the calculation of true incidence rates. Third, the absence of systematic data on critical clinical confounders severely limits our ability to perform multivariable adjustments. Because FAERS is a spontaneous reporting system heavily driven by consumer reports (92.3%), documentation of past medical history and chronic metabolic or cardiovascular comorbidities is overwhelmingly missing or inconsistently coded. Furthermore, FAERS lacks an “untreated” control cohort, relying instead on a background comparator of patients experiencing adverse events from other drugs. Consequently, we could not adjust for baseline cardiovascular risk factors, diabetes duration, smoking status, or concomitant medications. Fourth, although our study period (2022 Q2–2025 Q3) represents the entire post-marketing period currently available in FAERS for tirzepatide, this timeframe is relatively short. Longer observation periods would provide more stable safety signals and allow for the detection of late-emerging adverse events. Therefore, our findings should be interpreted purely as hypothesis-generating reporting patterns requiring validation in prospective, controlled cardiovascular outcome trials or large-scale electronic health record (EHR) studies.

## Conclusion

In this large-scale FAERS analysis using a concurrent full-database comparator, tirzepatide was associated with persistently lower reporting odds for heart failure across all sensitivity strata, including HCP-restricted analyses. No association was detected for atrial fibrillation, tachycardia, or ischemic events (acute myocardial infarction, angina, and myocardial ischemia) after accounting for reporter bias. Elevated reporting odds for tachycardia observed in weight-stratified analyses were confined to a small subset with documented weight (5.7%) and were not replicated in HCP reports. These findings reflect database reporting patterns influenced by channeling bias and competing adverse events, and do not prove clinical cardioprotection. This real-world evidence complements the safety database of tirzepatide and generates hypotheses for ongoing cardiovascular outcome trials.

## Data Availability

Publicly available datasets were analyzed in this study. This data can be found here: FDA Adverse Event Reporting System (FAERS) Public Dashboard and Quarterly Data Extract Files (https://fis.fda.gov/extensions/FPD-QDE-FAERS/FPD-QDE-FAERS.html). Further inquiries regarding the processed data can be directed to the corresponding authors.
